# HFE Gene Variants Modify the Association between Maternal Lead Burden and Infant Birthweight: A Prospective Birth Cohort Study in Mexico City, Mexico

**DOI:** 10.1186/1476-069X-9-43

**Published:** 2010-07-26

**Authors:** David Cantonwine, Howard Hu, Martha Maria Téllez-Rojo, Brisa N Sánchez, Héctor Lamadrid-Figueroa, Adrienne S Ettinger, Adriana Mercado-García, Mauricio Hernández-Avila, Robert O Wright

**Affiliations:** 1 Department of Environmental Health Sciences, University of Michigan School of Public Health, Ann Arbor, Michigan, USA; 2 Department of Environmental Health, Harvard School of Public Health, Boston, Massachusetts, USA; 3 Channing Laboratory, Department of Medicine, Brigham and Women's Hospital, Harvard Medical School, Boston, Massachusetts, USA; 4 Division of Statistics, Center for Evaluation Research and Surveys, National Institute of Public Health, Cuernavaca, Morelos, México; 5 Department of Biostatistics, University of Michigan School of Public Health, Ann Arbor, Michigan, USA; 6 Division of Environmental Health, Center for Population Health Research, National Institute of Public Health, Cuernavaca, Morelos, México; 7 Ministry of Health, México, Districto Federal, México

## Abstract

**Background:**

Neonatal growth is a complex process involving genetic and environmental factors. Polymorphisms in the hemochromatosis (*HFE*) iron regulatory genes have been shown to modify transport and toxicity of lead which is known to affect birth weight.

**Methods:**

We investigated the role of *HFE C282Y*, *HFE H63 D*, and transferrin *(TF) P570 S *gene variants in modifying the association of lead and infant birthweight in a cohort of Mexican mother-infant pairs. Subjects were initially recruited between 1994-1995 from three maternity hospitals in Mexico City and 411 infants/565 mothers had archived blood available for genotyping. Multiple linear regression models, stratified by either maternal/infant *HFE *or *TF *genotype and then combined with interaction terms, were constructed examining the association of lead and birthweight after controlling for covariates.

**Results:**

3.1%, 16.8% and 17.5% of infants (N = 390) and 1.9%, 14.5% and 18.9% of mothers (N = 533) carried the *HFE C282Y*, *HFE H63D*, and *TF P570 S *variants, respectively. The presence of infant *HFE H63 D *variants predicted 110.3 g (95% CI -216.1, -4.6) decreases in birthweight while maternal *HFE H63 D *variants predicted reductions of 52.0 g (95% CI -147.3 to 43.2). Interaction models suggest that both maternal and infant *HFE H63 D *genotype may modify tibia lead's effect on infant birthweight in opposing ways. In our interaction models, maternal *HFE H63 D *variant carriers had a negative association between tibia lead and birthweight.

**Conclusions:**

These results suggest that the *HFE H63 D *genotype modifies lead's effects on infant birthweight in a complex fashion that may reflect maternal-fetal interactions with respect to the metabolism and transport of metals.

## Background

Decreased birthweight has been established as a predictor of infant mortality, morbidity, developmental outcomes such as cognitive performance, and chronic disease into adulthood [1]. Both environmental and genetic factors contribute to the weight of an infant at birth. Environmental factors that have been associated with birth weight include, but are not limited to: maternal nutritional status [2], maternal infections [3], parity, and exposure to toxicants, such as lead [4]. Two recent population studies have estimated that approximately 50% of the variation in birthweight is due to heritable maternal/infant factors [5,6]. Genetic components which metabolize, respond to, or regulate environmental factors, such as lead exposure, would be strong candidates for studying gene-environment interactions.

Environmental exposure to lead [1] and iron deficiency [7,8] continue to contribute significantly to worldwide infant mortality and neurodevelopmental toxicity. Decreases in birth weight have been independently associated with increased lead exposure [4,9] and extremes in iron status [10,11]. These effects may be compounded when women are both iron deficient and exposed to lead during pregnancy, because lead absorption is upregulated during iron deficiency [12,13].

Iron uptake across cell membranes is complex and regulated by multiple proteins including transport proteins such as transferrin (*TF*), divalent metal transporter-1 (DMT-1), and ferroportin, their receptors, as well as regulatory proteins such as *HFE *and hepcidin[14]. The *HFE *gene is of particular interest in public health research because it contains two highly prevalent functional variants, *C282Y *and *H63 D *[15]. Several studies have indicated that *HFE *variants alone can be important modifiers of lead susceptibility to health outcomes [16,17] and these effects appear to be age dependent. For example, Wright et. al. showed that older adult carriers of either *HFE *variant genotype (*H63 D *or *C282Y*), had lower blood, patella bone, and tibia bone lead stores when compared to wildtype individuals [18]. Conversely, it was demonstrated that iron metabolism gene variants in both *HFE *and *TF *were associated with increased blood lead levels in Mexican children [19].

The aim of this study was to explore both the main gene and the interactive effects between variants in the iron regulatory protein gene *HFE *(*C282Y *and *H63D*), the iron transport protein gene *TF *(*P570S*), and biomarkers of neonatal lead exposure on infant birth weight. We hypothesized that: 1) maternal/infant *HFE *and *TF *variant genotypes would both independently and jointly modify infant birthweight; and, 2) *HFE *variant and *TF *variants could be protective against the negative effects of lead exposure on birth weight since they could foster adequate iron stores that would compete for common transport receptors.

## Methods

### Sample population

Maternal/infant pairs were recruited between 1994 and 1995 from three hospitals in Mexico City which serve low-to-moderate income populations as part of a clinical trial to assess calcium supplementation effects on maternal bone lead mobilization during lactation using methods previously described [4,20]. Of the original cohort of 617 mother-infant pairs enrolled, there were 411 infants and 565 mothers who had archived blood available for genotyping for this analysis.

The study protocol was approved by the Ethics Committee of the National Institute of Public Health of Mexico, the participating hospitals, the Brigham and Women's Hospital, and the Harvard School of Public Health. All participating mothers signed a written consent form, received a detailed explanation of the study intent and research procedures, as well as counseling on how to reduce environmental lead exposure.

### Anthropometric measurements

Neonates were weighed at delivery by experienced obstetric nurses using calibrated beam scales (Oken, Model TD16, Naucalpan, Mexico) read to the nearest 10 grams. Maternal anthropometric measures were collected by our trained project personnel and standardized according to the technique described by Habicht [21]. These standardization exercises were performed until the project staff reached imprecision errors equal to or below those reported by Lohman and coworkers [22]. Accepted technical errors were 0.3 cm for arm circumference and 0.22 cm for height.

### Blood lead measurements

Umbilical cord blood samples were collected in trace metal-free tubes at delivery. Blood samples were analyzed using an atomic absorption spectrometry instrument (Perkin-Elmer 3000, Chelmsford, MA, USA) at the metals laboratory of the American British Cowdray Hospital in Mexico City. External blinded quality-control samples were provided throughout the study period by the Maternal and Child Health Bureau and the Wisconsin State Laboratory of Hygiene Cooperative Blood Lead Proficiency Testing Program. Our laboratory maintained acceptable precision and accuracy over the study period [correlation = 0.98; mean difference = 0.71 μg/dL; SD = 0.68].

### Maternal bone lead measurements

Maternal bone lead was measured non-invasively using a spot-source ^109^Cd K-XRF instrument constructed at Harvard University and installed in a research facility in the American British Cowdray Medical Center. For this study, 30-minute *in vivo *maternal bone lead measurements were taken within one month of delivery at two bone sites, the mid-tibial shaft (cortical bone) and the patella (trabecular bone). The physical principles, technical specifications, and validation of this and other similar K-XRF instruments have been described in detail elsewhere [23].

### Genotyping methods

DNA extraction and genotyping were performed in The Harvard-Partners Center for Genetics and Genomics. High-molecular-weight DNA was extracted with commercially available PureGene Kits (Gentra Systems, Minneapolis, MN) from the white blood cells of archived maternal and umbilical cord blood samples. Genotyping for the hemochromotosis *HFE C282Y *(RS1800562), *HFE H63 D *(RS1799945) and transferrin *TF P570 S *(RS1049296) variants was performed using Sequenom MALDI-TOF (Matrix-assisted laser desorption ionization - time of flight) mass spectrometry according to the methods that have been described earlier [19].

### Statistical analyses

Descriptive statistics and identification of outliers, using the generalized extreme studentized deviation method [24] were performed. Distribution of *HFE *and *TF *alleles and genotypes were examined and frequencies were tested using a chi-square statistic to compare observed and expected counts according to principles of Hardy-Weinberg equilibrium. Due to the low prevalence of *HFE C282Y *variants in this population, this variant was excluded from further analyses. Individuals homozygous for *HFE H63 D *(N = 4 infants, N = 3 mother) and those who were compound heterozygotes with *HFE C282Y *(N = 2 infants, N = 1 mother) were removed from analysis due to population studies that demonstrate those individuals may have significantly increased iron levels when compared to carrier individuals (Jackson et al. 2001). Remaining individuals with heterozygous variant genotypes were compared separately to participants with wild type genotypes for each of the two remaining variants (*HFE H63 D and TF 570S*).

Demographic characteristics and lead levels by genotype were examined and mean differences were tested by chi-square or Student's *t*-test (2-tailed) for continuous and categorical variables as appropriate. Potential non-linearity between continuous predictor variables and birth weight was explored by plotting results from generalized additive models. Resulting non-linear associations were controlled for as covariates, in multiple linear regression models which included either the maternal *HFE H63 D *or *TF P570 S *genotype, by adding a squared term for maternal age and dummy variables for maternal education (years of education: <8, 8 -11, 11>). Multiple linear regression was then used to model the relationship between birthweight, maternal and infant *HFE H63 D *and *TF P570 S *genotypes, and biomarkers of lead exposure, after controlling for potential confounding variables. The potential confounding variables considered in our models were based on biologic plausibility (maternal age at delivery (years), maternal education (years), cigarette smoking during pregnancy (yes/no), gestational age (weeks), infant gender (female gender as reference group), and parity (total number of live births), or those significantly associated with birthweight (p < 0.1) in bivariate analysis (maternal postpartum arm circumference (cm)( (which served as proxy for gestational weight gain), maternal hemoglobin at 1 month post-partum, and marital status (single/partnered.) To examine the potential modifying effect of the variants, we initially ran separate multiple linear regression models stratified by maternal or infant genotype. On the basis of the differences in effect estimates of tibia bone lead on birthweight in stratified models, we fitted multiple linear regression models that included an interaction term between the genotype and tibia bone lead.

Regression diagnostics were performed on all models to evaluate multicollinearity and violations of the linear regression model assumptions. Data were analyzed using SAS 9.1, SAS Institute Inc. Cary, NC, 2002-2003 and R 2.9.1, (The R Foundation for Statistical Computing, Boston, MA 2007.

## Results

Our final study population included 533 genotyped mothers of whom 1.9%, 14.5%, and 18.9% carried the *HFE C282Y*, *HFE H63D*, and *TF P570 S *variant, respectively. Additionally, 390 genotyped children were included and 3.1%, 16.8%, and 17.5% carried the *HFE C282Y, HFE H63D*, and *TF P570 S *variants, respectively (Table 1). All genotype distributions were found to conform to Hardy-Weinberg equilibrium expectations. After taking into consideration both maternal and infant *HFE H63 D *genotype status, 20 (5.4%) mothers carried the *HFE H63 D *variant while their infants were wildtype, 32 (8.6%) mothers were wildtype while their infants carried the *HFE H63 D *variant, and 40 (10.8%) were both heterozygous for *HFE H63D*.

**Table 1:** Maternal and Infant *HFE *and *TF *Genotype Frequencies

Mother			Infant		
**Genotype**	**Number**	**%**	**Genotype**	**Number**	**%**

**HFE (C282Y) **			**HFE (C282Y) **		

C282 Wildtype (CC)	523	98.1	C282 Wildtype (CC)	378	96.9

C282Y Heterozygous (CY)	10	1.9	C282Y Heterozygous (CY)	10	3.1

C282Y Homozygous (YY)	0	0	C282Y Homozygous (YY)	0	0

Hardy-Weinberg equilibrium: χ^2 ^= 0.05, p = 0.83			Hardy-Weinberg equilibrium: χ^2 ^= 0.10, p = 0.76		

					

**HFE (H63D) **			**HFE (H63D) **		

H63 Wildtype (HH)	452	85.0	H63 Wildtype (HH)	319	82.2

H63 D Heterozygous (HD)	77	14.5	H63 D Heterozygous (HD)	65	16.8

H63 D Homozygous (DD)	3	0.5	H63 D Homozygous (DD)	4	1.0

Hardy-Weinberg equilibrium: χ^2 ^= 0.02, p = 0.87			Hardy-Weinberg equilibrium: χ^2 ^= 0.11, p = 0.74		

					

**TF (P570S) **			**TF (P570S) **		

P570 Wildtype (PP)	429	80.9	P570 Wildtype (PP)	319	82.0

P570 S Heterozygous (PS)	100	18.9	P570 S Heterozygous (PS)	68	17.5

P570 S Homozygous (SS)	1	0.2	P570 S Homozygous (SS)	2	0.5

Hardy-Weinberg equilibrium: χ^2 ^= 3.81, p = 0.05			Hardy-Weinberg equilibrium: χ^2 ^= 0.65, p = 0.42		

Table 2 shows the distribution of lead biomarkers and covariates stratified by maternal and infant *HFE H63 D *and *TF P570 S *genotypes. Mean infant birth weight was slightly lower in infant *HFE H63 D *carriers when compared to infant *HFE H63 *wild-type individuals. There were no significant differences between maternal *HFE H63 D *variants and maternal wild-type individuals. Maternal hemoglobin at one month post partum were significantly increased in maternal genotype *TF P570 S *variants when compared to maternal *TF P570 *wild-type individuals respectively.

**Table 2:** Study population characteristics by Maternal *HFE H63 D *and *TF P570 S *genotype

	H63 Wildtype	H63 D Variant	TF Wildtype	TF Variant
Age (years)	24.5 (5.0)	23.9 (5.2)	24.4 (5.0)	24.4 (5.2)

Education (years)	9.3 (3.0)	9.3 (2.9)	9.4 (3.0)	9.3 (3.1)

Arm circumference	26.4 (2.7)	26.2 (2.5)	26.3 (2.7)	26.6 (2.7)

Smoking during pregnancy	4.7%	2.7%	4.7%	3.0%

Primiparity	43.6%	49.3%	44.3%	41.4%

Hemoglobin 1 month (g/dL)	13.6 (1.5)	13.6 (1.6)	13.5 (1.5)	13.8 (1.4)**

Marital Status (% Married)	65.5%	61.3%	64.5%	64.7%

Tibia Bone Lead (ug/g)	10.1 (9.7)	8.7 (9.3)	10.1 (9.8)	9.4 (9.0)

Gestational Age (days)	39.3 (1.4)	39.1 (1.4)	39.2 (1.4)	39.4 (1.3)

Birthweight (grams)	3162.2 (418.1)	3091.7 (415.8)	3156.6 (420.4)	3134.5 (404.9)

Sex (% Male)	54.9%	50.7%	54.7%	54.6%

Cord Blood Lead (μg/dL)	6.6 (3.5)	6.3 (4.2)	6.5 (3.5)	6.9 (1.3)


*P(< 0.1), **P(< 0.05)

There were 59 (12.6%) infants with cord blood lead levels exceeding 10 μg/dL. Additionally, 147 (26.0%) mothers were anemic based on criteria for evaluating lactating women living at an altitude of 7,000 - 7,999 feet above sea level [25]. Cord blood lead failed to predict a significant association with birthweight after controlling for covariates of interest (Table 3). A one-unit change in tibia bone lead predicted a decrease of 4.4 g (95%CI: -7.9, -0.9) in birthweight after controlling for covariates of interest. Using the lowest quartile of tibia lead as the reference group, the highest quartile of tibia lead predicted a 95.4 gram (95%CI: -189.9, -0.8) gram decrease in birth weight and the trend across quartiles had a p-value of 0.06.

**Table 3:** Adjusted parameter estimates for birthweight by biological markers of lead exposure in separate linear regression models^a^

Variable	N	β	P-value	95% CI
Cord Blood Lead (μg/dL)	464	-31.1	0.41	-105.4, 43.3

Maternal Blood at Delivery (μg/dL)	526	9.3	0.80	-64.2, 82.9

				

Tibia Lead Continuous (μg/g)	538	-4.4	0.01	-7.9, -0.9

				

Tibia Lead Quartiles				

Q1 (< 1 - 4.1)	137	Reference		

Q2 (4.1 - 9.2)	137	17.2	0.72	-75.6, 110.1

Q3 (9.2 - 15.4)	137	-19.1	0.69	-112.1, 73.9

Q4 (15.4 - 43.2)	137	-95.4	0.05	-189.9, -0.8

		P-Trend	0.06	

^a^All models are adjusted for maternal age, years of maternal education, infant gender, maternal arm circumference, gestational age, smoking status during pregnancy, marital status, maternal hemoglobin one month post-partum, and parity

Number - N, Coefficient - β, Confidence Interval - CI.

Results from generalized additive models indicated that gestational age had a slight non-linear association with birthweight. Additionally, in models with the maternal *HFE H63 D *or *TF P570 S *variants, maternal age and maternal education had significant non-linear relationships. To control for this effect, any models including the maternal *HFE H63 D *or *TF P570 S *included additional covariates: maternal age squared and tertiles of maternal education.

Table 4 lists the main gene effects for maternal/infant *HFE H63 D *and *TF P570 S *upon infant birthweight. After controlling for tibia bone lead and potential confounding variables of birth weight in multivariate analysis (maternal age at delivery in years, maternal years of education, maternal arm circumference, cigarette smoking during pregnancy (yes/no), gestational age in weeks, infant sex, maternal hemoglobin at 1 month post-partum, and parity), the presence of the infant and maternal variant *HFE H63 D *gene predicted a 129.5 g (95% CI: -236.4, -22.6) and 53.7 g (95% CI: -148.9, 41.5) decrease in birthweight respectively. The presence of both a maternal and infant *HFE H63 D *variant genotype predicted a decrease of 176.9 g (95% CI: -318.6 to -35.3) in birthweight. Main effects of maternal and infant transferrin variant genotypes on birthweight were non-significant. When both compound heterozygotes and *HFE H63 D *homozygote individuals were included as "carriers (i.e. recoded as HFE = 1 versus HFE = 0 for wildtype) presence of the infant and maternal variant *HFE H63 D *gene predicted a 89.6 g (95% CI: -190.9, 11.9) and 49.4 g (95% CI: -142.1, 43.2) decrease in birthweight respectively.

**Table 4:** Adjusted parameter estimates for the association of *HFE H63 D and TF P570 S *genotype status and birthweight, in separate linear regression models^a^

Model	Variable	N	β	95% CI
1	Infant *HFE H63 D *Genotype	367	-129.5**	-236.4, -22.6

2	Infant *TF P570 S *Genotype	371	34.9	-68.3,138.2

				

3	Maternal *HFE H63 D *Genotype^b^	502	-53.7	-148.9, 41.5

4	Maternal *TF P570 S *Genotype^b^	503	-62.6	-148.5, 23.4

				

5	H63_Inf. _Wildtype/H63_Mat._Wildtype	Referent (N = 260)		

	H63_Inf. _Wildtype/H63D_Mat._Variant	16	94.7	-99.0, 288.4

	H63D_Inf. _Variant/H63_Mat._Wildtype	24	-77.4	-240.9, 86.1

	H63D_Inf. _Variant/H63D_Mat._Variant	32	-176.9**	-318.6, -35.3

				

6	TF_Inf. _Wildtype/TF_Mat._Wildtype	Referent (N = 252)		

	TF_Inf. _Wildtype/TF_Mat._Variant	31	-63.2	-207.2, 80.8

	TF_Inf. _Variant/TF_Mat._Wildtype	27	66.9	-88.6, 222.3

	TF_Inf. _Variant/TF_Mat._Variant	27	-36.4	-190.1,117.2

^a^All models are adjusted for maternal age, years of maternal education, infant gender, maternal arm circumference, gestational age, smoking status during pregnancy, marital status, maternal tibia lead, maternal hemoglobin at 1 month post-partum, and parity.

^b^All models additionally adjusted for maternal age^2^, and dummy variables for maternal education (years of education: <8, 8 -11, 11>) to account for the non-linear nature of these covariates.

** P-value < 0.05, * P-value < 0.1

Infant - Inf., Maternal - Mat. Number - N, Coefficient - Coef., Confidence Interval - CI

We next examined the potential effect modification by *HFE *and *TF *genotype on the adverse relationship between maternal bone lead levels and birthweight listed in table 5. We found that the maternal *HFE H63 D *genotype may modify the relationship between tibia lead and birthweight by enhancing the negative effect in maternal *HFE H63 D *variants. The coefficient for the interaction term between tibia lead and maternal *HFE H63 D *genotype was -10.3 (p = 0.05). This relationship persisted when we modeled infant-maternal genotype interactions as dummy variables; maternal *HFE H63 D *variants who gave birth to infant *HFE H63 *wildtypes had an enhanced negative effect of tibia lead on birthweight with an interaction term of -28.7 (P = 0.003). Effect modification by infant *HFE H63 D *and *TF P570 S *status on the tibia lead-birthweight relationship were not significant, but suggested a positive direction for effect modification. There were no significant interactions between maternal blood at delivery or cord blood and genotype status (data not shown.)

**Table 5:** Adjusted regression coefficients for effect modification of HFE or TF genotype upon tibia lead's adverse association with birthweight^a^

Model	Genotype	Genotype Variant	Tibia Lead	Interaction Term
1	Infant *HFE H63D*	-127.5**	-6.5**	4.5

2	Infant *TF P570S*	34.9	-5.8**	0.9

				

3	Maternal *HFE H63 D *^b^	-62.7	-3.0	-10.3**

4	Maternal *TF P570 S *^b^	-60.8	-5.7**	6.8

				

5	Maternal/Infant *HFE H63 D *Interactions			

	H63_Inf. _Wildtype/H63_Mat._Wildtype	Referent		

	H63_Inf. _Wildtype/H63D_Mat._Variant	143.0	-4.5*	-28.7**

	H63D_Inf. _Variant/H63_Mat._Wildtype	-64.2		6.2

	H63D_Inf. _Variant/H63D_Mat._Variant	-177.2**		-6.0

				

6	Maternal/Infant *TF P570 S *Interactions			

	TF_Inf. _Wildtype/TF_Mat._Wildtype	Referent		

	TF_Inf. _Wildtype/TF_Mat._Variant	-51.7	-7.2**	10.3

	TF_Inf. _Variant/TF_Mat._Wildtype	66.7		-4.5

	TF_Inf. _Variant/TF_Mat._Variant	-40.5		9.3

^a^All models are adjusted for maternal age, years of maternal education, infant gender, maternal arm circumference, gestational age, smoking status during pregnancy, maternal hemoglobin one month post-partum, and parity.

^b^All models additionally adjusted for maternal age^2^, and dummy variables for maternal education (years of education: <8, 8 -11, 11>) to account for the non-linear nature of these covariates.

* P-value < 0.1, ** P-value < 0.05

Infant - Inf., Maternal - Mat.

## Discussion

Our results demonstrate that the infant *HFE H63 D *variant genotype predicts a decrease in birth weight. In multiple linear regression models, the infant *HFE H63 D *allele predicted a decrease of 129.5 grams (95%CI: -236.4, -22.6) in infant birth weight after controlling for covariates of interest, including tibia lead levels. The combined effects of both the maternal and infant *HFE H63 D *variant genotype predicted a greater decrease in birthweight of 176.9 grams (95%CI: -318.6, -35.3). This would suggest that maternal-fetal genotypes are interactive for this gene, an interesting and unique finding. Finally, the maternal *HFE H63 D *variant allele predicted enhanced negative effects of maternal bone lead on birthweight. To our knowledge this study is the first to observe effect modification of the association between lead exposure and birth weight by *HFE *genotype status.

Because iron is a critical nutrient, has been related to lead absorption, and has been linked to birthweight previously, studies of iron metabolism gene variants are logical candidates to either directly impact birthweight or modify the effect of lead on birthweight. Our study focused on two well known functional variants within iron metabolism genes. The independent effects of *HFE *on birth weight have only been studied in one other study [26]. In Maier and colleagues' study, very low birth weight infants (< 1500 g) were assessed for *HFE C282Y *genotype and transferrin saturation. Although the study observed no association between *HFE C282Y *genotype, transferrin saturation and very low birth weight, it should be noted that only six infants were heterozygous for the *HFE C282Y *mutation so the study was very underpowered to test this hypothesis. Our study differs from Maier et. al. in that we had no very low birth weight infants and instead chose to look at the continuous measure of birth weight.

A mechanistic role for the interaction between *HFE*, lead, and birth weight may lie both within the role of iron status as a modifier of lead absorption and the role of iron as a toxicant. Previous studies on lead uptake during iron deficient conditions have demonstrated that both lead and iron compete for binding to the DMT-1 transporter [27,28]. On the apical plasma membrane of syncytiotrophoblastic cells in human placenta, *HFE *associates with the transferrin receptor and on the basal side with ferroprotin and DMT-1, suggesting a potential role in iron transport across the placenta [29-33]. Disregulation in HFE has been shown to increase DMT-1 mediated intestinal uptake of iron, but studies are lacking for these effects upon lead absorption or in placental transfer [34-37]. In human epidemiological studies, presence of the HFE H63 D variant resulted in higher child blood lead levels [19]. Taken together these previous studies could explain, in part, the increased risk of low birthweight for infants with the H63 D allele though this effect may not be limited to changes in lead absorption/metabolism since excess iron itself is potentially toxic.

Previous research has also shown a U-shaped relation where both maternal iron deficiency and iron excess can increase risk of preterm delivery and decrease infant birthweight [38-42]. Researchers have shown that in response to low iron and anemic conditions in early and late pregnancy changes in placental morphology can occur. These changes manifest as larger weight placentas with observed increases in villous volume and surface areas of the capillaries involved in gas exchange, which thought to be a response to hypoxic conditions that would limit growth [43-46]. Alternatively, the production of reactive oxygen species resulting from reactive iron species, like unbound iron, is thought to be the major mechanistic contributor to the damage done by iron excess [38,47,48]. Exposure to lead has also been shown to disrupt the balance between reactive oxygen species and antioxidant cellular defenses [49,50]. The H63 D allele may act both by increasing iron to subclinical toxic levels and by increasing lead. We hypothesize that a disruption in the delicate balance of pro-oxidant/antioxidant molecules either through excess/deficiency of iron and exposure to lead could impair birth weight. We speculate that these effects could be enhanced when both the mother and infant carry the variant allele potentially due to a similar mechanistic pathway. The enhancement of lead's negative effects by maternal *HFE H63 D *variant status indicated by our results may also be mediated through such a mechanism. Further research is needed in order to gain a greater understanding of how biologically useful metals interact with toxic metals and how these interactions may modify health effects.

Many past gene-environment epidemiological studies which focus on reproductive outcomes tend only to report either maternal or fetal genotype results while neglecting the combined maternal-fetal genetic susceptibility. In our study we found that while infants who carry the H63 D variant are adversely associated with birthweight, it was the infant H63 D carriers born to mothers which are also H63 D carriers that had the greatest adverse association with birthweight. Several other recent studies have observed similar relationships where maternal/infant variant-variant carriers show the greatest extremes in risk for adverse birth outcome [51-54].

As with any study, there are limitations. In our study we did not have a direct measure of maternal iron status during pregnancy and instead used maternal hemoglobin at 1 month post-partum as an indirect measure. Hemoglobin will reflect severe iron deficiency but cannot detect iron deficiency without anemia. While research has indicated that maternal hemoglobin during pregnancy can predict decreases in birthweight, it has been extensively discussed that a single biomarker of iron status is insufficient in determining true iron status [8]. We were also unable to take measures of ferritin, hemoglobin, mean corpuscular volume, free erythrocyte protoporphyrin, and other markers of iron status during pregnancy due to the original study design, since recruitment began at delivery. Without more extensive measures of iron status we were unable to assess iron deficiency without anemia or iron excess which have both been linked with decreased birthweight in numerous studies [12,13,38,42]. As a surrogate measure for iron status we used hemoglobin measures at one month post partum which was significantly associated with a decrease in birthweight in our regression models suggesting a role for iron metabolism in predicting birthweight. Previous studies have indicated that iron levels are slightly elevated in heterozygous carriers of either *HFE C282Y *or *HFE H63 D *genotypes [55]. In our study maternal carriers of *HFE H63 D *variant genotypes did not have significantly higher one month post-partum hemoglobin measures (13.7 (1.6) μg/dL) when compared to wildtype individuals (13.5 (1.5) μg/dL, P = 0.13), but we cannot determine if their body iron stores were higher without measures of ferritin or transferring saturation. Finally, given the small sample size to test for interactions, these results should be considered preliminary.

## Conclusions

In summary, we found that infants (and mother-infant pairs) who both carry the *HFE H63 D *variant have, on average, lower birth weights. Additionally, infant *HFE H63 D *variants may modify the negative effects of lead biomarkers on birth weight by decreasing lead effects. Conversely, mothers who carry an *HFE H63 D *variant may enhance the negative effects of maternal bone lead exposure on birthweight which may arise due to increased oxidative damage during fetal development.

## List of Abbreviations

DMT-1: Divalent Metal Transporter 1; HFE: Hemochromatosis; K-XRF: K-X-ray fluorescence; MLR: Multiple Linear Regression; ROS: Reactive Oxygen Species; STB: Syncytiotrophoblastic; Tfr: transferrin receptor; μg/dL: micrograms per deciliter; μg/g: micrograms per gram; VLBW: very low birth weight.

## Competing interests

The authors declare that they have no competing interests.

## Authors' contributions

Authors contributed to the article as follows: DC conducted the literature review, designed and conducted the statistical analysis, and wrote the manuscript. HH designed the parent study, directed its implementation, and provided critical feedback on the manuscript. MMTR designed the parent study, directed its implementation, and provided feedback on the manuscript.BNS helped to direct the statistical analysis/interpretation for the study and provided feedback on the Methods section. HLF helped to supervise the field activities and provided feedback on the manuscript. ASE helped supervise the field activities and primary data collection and provided feedback on the manuscript. AMG helped supervise the field activities and primary data collection. MHA designed the parent study and directed its implementation. ROW assisted in the design the parent study, directed its implementation, and provided feedback on the manuscript. All authors read and approved the final manuscript.

## References

[B1] Tong S Baghurst P McMichael A Birthweight and cognitive development during childhood J Paediatr Child Health 2006 42 98 103 10.1111/j.1440-1754.2006.00805.x 16509907

[B2] Ramakrishnan U Nutrition and low birth weight: from research to practice Am J Clin Nutr 2004 79 17 21 14684392 10.1093/ajcn/79.1.17

[B3] Rees S Harding R Brain development during fetal life: influences of the intra-uterine environment Neuroscience letters 2004 361 111 114 10.1016/j.neulet.2004.02.002 15135906

[B4] Gonzalez-Cossio T Peterson KE Sanin LH Fishbein E Palazuelos E Aro A Hernandez-Avila M Hu H Decrease in birth weight in relation to maternal bone-lead burden Pediatrics 1997 100 856 862 10.1542/peds.100.5.856 9346987

[B5] Lunde A Melve KK Gjessing HK Skjaerven R Irgens LM Genetic and Environmental Influences on Birth Weight, Birth Length, Head Circumference, and Gestational Age by Use of Population-based Parent-Offspring Data Am J Epidemiol 2007 165 7 734 41 10.1093/aje/kwk107 17311798

[B6] Dunger DB Petry CJ Ong KK Genetic variations and normal fetal growth Horm Res 2006 65 34 40 10.1159/000091504 16612112

[B7] Walker SP Wachs TD Gardner JM Lozoff B Wasserman GA Pollitt E Carter JA Child development: risk factors for adverse outcomes in developing countries Lancet 2007 369 145 157 10.1016/S0140-6736(07)60076-2 17223478

[B8] WHO C Assessing the Iron Status of Populations 2004 Organization WH. Geneva: WHO/CDC Joint Report

[B9] Jelliffe-Pawlowski LL Miles SQ Courtney JG Materna B Charlton V Effect of magnitude and timing of maternal pregnancy blood lead (Pb) levels on birth outcomes Journal of Perinatology 2006 26 154 162 10.1038/sj.jp.7211453 16453008

[B10] Lozoff B Georgieff MK Iron deficiency and brain development Semin Pediatr Neurol 2006 13 158 165 10.1016/j.spen.2006.08.004 17101454

[B11] Milman N Iron and pregnancy--a delicate balance Ann Hematol 2006 85 559 565 10.1007/s00277-006-0108-2 16691399

[B12] Wright RO Tsaih SW Schwartz J Wright RJ Hu H Association between iron deficiency and blood lead level in a longitudinal analysis of children followed in an urban primary care clinic J Pediatr 2003 142 9 14 10.1067/mpd.2003.mpd0344 12520247

[B13] Wolf AW Jimenez E Lozoff B Effects of iron therapy on infant blood lead levels J Pediatr 2003 143 789 795 10.1067/S0022-3476(03)00540-7 14657829

[B14] Rivers CA Barton JC Gordeuk VR Acton RT Speechley MR Snively BM Leiendecker-Foster C Press RD Adams PC McLaren GD Association of ferroportin Q248 H polymorphism with elevated levels of serum ferritin in African Americans in the Hemochromatosis and Iron Overload Screening (HEIRS) Study Blood Cells, Molecules, and Diseases 2007 38 247 252 10.1016/j.bcmd.2006.12.002 PMC3727273 17276706

[B15] Hanson EH Imperatore G Burke W HFE gene and hereditary hemochromatosis: a HuGE review. Human Genome Epidemiology Am J Epidemiol 2001 154 193 206 10.1093/aje/154.3.193 11479183

[B16] Onalaja AO Claudio L Genetic susceptibility to lead poisoning Environmental Health Perspectives 2000 108 23 28 10.2307/3454630 10698721 PMC1637782

[B17] Wang FT Hu H Schwartz J Weuve J Spiro AS Sparrow D Nie H Silverman EK Weiss ST Wright RO Modifying effects of the HFE polymorphisms on the association between lead burden and cognitive decline Environ Health Perspect 2007 115 1210 1215 10.1289/ehp.9855 17687449 PMC1940090

[B18] Wright RO Silverman EK Schwartz J Tsaih SW Senter J Sparrow D Weiss ST Aro A Hu H Association between hemochromatosis genotype and lead exposure among elderly men: the normative aging study Environ Health Perspect 2004 112 746 750 15121519 10.1289/ehp.6581 PMC1241970

[B19] Hopkins MR Ettinger AS Hernandez-Avila M Schwartz J Tellez-Rojo MM Lamadrid-Figueroa H Bellinger D Hu H Wright RO Variants in iron metabolism genes predict higher blood lead levels in young children Environ Health Perspect 2008 116 1261 1266 10.1289/ehp.11233 18795173 PMC2535632

[B20] Hernandez-Avila M Gonzalez-Cossio T Hernandez-Avila JE Romieu I Peterson KE Aro A Palazuelos E Hu H Dietary calcium supplements to lower blood lead levels in lactating women: a randomized placebo-controlled trial Epidemiology 2003 14 206 212 10.1097/00001648-200303000-00015 12606887

[B21] Habicht JP [Standardization of quantitative epidemiological methods in the field] Boletin de la Oficina Sanitaria Panamericana 1974 76 375 384 4277063

[B22] Lohman Roche Martorell Anthropometric standarization reference manual Human Kinetics Book 1988 Champaign: Human Kinetics Publishers

[B23] Aro AC Todd AC Amarasiriwardena C Hu H Improvements in the calibration of 109Cd K x-ray fluorescence systems for measuring bone lead in vivo Physics in medicine and biology 1994 39 2263 2271 10.1088/0031-9155/39/12/009 15551552

[B24] Rosner B Percentage Points for a Generalized ESD Many-Outlier Procedure Technometrics 1983 25 165 172 10.2307/1268549

[B25] CDC Recommendations to prevent and control iron deficiency in the United States MMWR Recomm Rep 1998 47 1 29 9563847

[B26] Maier RF Witt H Buhrer C Monch E Kottgen E HFE gene mutation and transferrin saturation in very low birthweight infants Archives of disease in childhood 1999 81 F144 145 10448186 10.1136/fn.81.2.f144 PMC1720994

[B27] Bannon D Portnoy ME Olivi L Lees PS Culotta VC Bressler JP Uptake of lead and iron by divalent metal transporter 1 in yeast and mammalian cells Biochem Biophys Res Commun 2002 295 978 984 10.1016/S0006-291X(02)00756-8 12127992

[B28] Bressler JP Olivi L Cheong JH Kim Y Bannona D Divalent metal transporter 1 in lead and cadmium transport Annals of the New York Academy of Sciences 2004 1012 142 152 10.1196/annals.1306.011 15105261

[B29] Georgieff MK Wobken JK Welle J Burdo JR Connor JR Identification and localization of divalent metal transporter-1 (DMT-1) in term human placenta Placenta 2000 21 8 799 804 10.1053/plac.2000.0566 11095929

[B30] Gruper Y Bar J Bacharach E Ehrlich R Transferrin receptor co-localizes and interacts with the hemochromatosis factor (HFE) and the divalent metal transporter-1 (DMT1) in trophoblast cells J Cell Physiol 2005 204 901 912 10.1002/jcp.20349 15880641

[B31] Bastin J Drakesmith H Rees M Sargent I Townsend A Localisation of proteins of iron metabolism in the human placenta and liver Br J Haematol 2006 134 532 543 10.1111/j.1365-2141.2006.06216.x 16856887

[B32] Parkkila S Waheed A Britton RS Bacon BR Zhou XY Tomatsu S Fleming RE Sly WS Association of the transferrin receptor in human placenta with HFE, the protein defective in hereditary hemochromatosis Proc Natl Acad Sci USA 1997 94 13198 13202 10.1073/pnas.94.24.13198 9371823 PMC24286

[B33] Ganz T Molecular control of iron transport J Am Soc Nephrol 2007 18 394 400 10.1681/ASN.2006070802 17229910

[B34] Garrick MD Dolan KG Horbinski C Ghio AJ Higgins D Porubcin M Moore EG Hainsworth LN Umbreit JN Conrad ME DMT1: a mammalian transporter for multiple metals Biometals 2003 16 1 41 54 10.1023/A:1020702213099 12572663

[B35] Fleming RE Migas MC Zhou X Jiang J Britton RS Brunt EM Tomatsu S Waheed A Bacon BR Sly WS Mechanism of increased iron absorption in murine model of hereditary hemochromatosis: increased duodenal expression of the iron transporter DMT1 Proc Natl Acad Sci USA 1999 96 3143 3148 10.1073/pnas.96.6.3143 10077651 PMC15909

[B36] Zoller H Koch RO Theurl I Obrist P Pietrangelo A Montosi G Haile DJ Vogel W Weiss G Expression of the duodenal iron transporters divalent-metal transporter 1 and ferroportin 1 in iron deficiency and iron overload Gastroenterology 2001 120 1412 1419 10.1053/gast.2001.24033 11313311

[B37] Rolfs A Bonkovsky HL Kohlroser JG McNeal K Sharma A Berger UV Hediger MA Intestinal expression of genes involved in iron absorption in humans Am J Physiol Gastrointest Liver Physiol 2002 282 G598 607 11897618 10.1152/ajpgi.00371.2001

[B38] Casanueva E Viteri FE Iron and oxidative stress in pregnancy Journal of Nutrition 2003 133 1700S 1708S 12730487 10.1093/jn/133.5.1700S

[B39] Swain S Singh S Bhatia BD Pandey S Krishna M Maternal hemoglobin and serum albumin and fetal growth Indian pediatrics 1994 31 777 782 7890339

[B40] Ronnenberg AG Wood RJ Wang X Xing H Chen C Chen D Guang W Huang A Wang L Xu X Preconception hemoglobin and ferritin concentrations are associated with pregnancy outcome in a prospective cohort of Chinese women The Journal of nutrition 2004 134 2586 2591 15465752 10.1093/jn/134.10.2586

[B41] Lee HS Kim MS Kim MH Kim YJ Kim WY Iron status and its association with pregnancy outcome in Korean pregnant women European journal of clinical nutrition 2006 60 1130 1135 10.1038/sj.ejcn.1602429 16639418

[B42] Lao TT Tam KF Chan LY Third trimester iron status and pregnancy outcome in non-anaemic women; pregnancy unfavourably affected by maternal iron excess Hum Reprod 2000 15 1843 1848 10.1093/humrep/15.8.1843 10920115

[B43] Hindmarsh PC Geary MP Rodeck CH Jackson MR Kingdom JC Effect of early maternal iron stores on placental weight and structure Lancet 2000 356 719 723 10.1016/S0140-6736(00)02630-1 11085691

[B44] Howe DT Wheeler T Osmond C The influence of maternal haemoglobin and ferritin on mid-pregnancy placental volume Br J Obstet Gynaecol 1995 102 213 219 7794845 10.1111/j.1471-0528.1995.tb09096.x

[B45] Kadyrov M Kosanke G Kingdom J Kaufmann P Increased fetoplacental angiogenesis during first trimester in anaemic women Lancet 1998 352 1747 1749 10.1016/S0140-6736(98)02069-8 9848351

[B46] Singla PN Tyagi M Kumar A Dash D Shankar R Fetal growth in maternal anaemia J Trop Pediatr 1997 43 89 92 10.1093/tropej/43.2.89 9143178

[B47] Lund EK Fairweather-Tait SJ Wharf SG Johnson IT Chronic exposure to high levels of dietary iron fortification increases lipid peroxidation in the mucosa of the rat large intestine The Journal of nutrition 2001 131 2928 2931 11694620 10.1093/jn/131.11.2928

[B48] Srigiridhar K Nair KM Subramanian R Singotamu L Oral repletion of iron induces free radical mediated alterations in the gastrointestinal tract of rat Molecular and cellular biochemistry 2001 219 91 98 10.1023/A:1011023111048 11354259

[B49] Casado MF Cecchini AL Simao AN Oliveira RD Cecchini R Free radical-mediated pre-hemolytic injury in human red blood cells subjected to lead acetate as evaluated by chemiluminescence Food Chem Toxicol 2007 45 945 952 10.1016/j.fct.2006.12.001 17250942

[B50] El-Sayed IH Lotfy M El-Khawaga OA Nasif WA El-Shahat M Prominent free radicals scavenging activity of tannic acid in lead-induced oxidative stress in experimental mice Toxicology and industrial health 2006 22 157 163 10.1191/0748233706th256oa 16786837

[B51] Liang M Wang X Li J Yang F Fang Z Wang L Hu Y Chen D Association of combined maternal-fetal TNF-alpha gene G308A genotypes with preterm delivery: a gene-gene interaction study J Biomed Biotechnol 2010 396184 20224765 10.1155/2010/396184 PMC2836175

[B52] Qin X Wu Y Wang W Liu T Wang L Hu Y Chen D Low organic solvent exposure and combined maternal-infant gene polymorphisms affect gestational age Occup Environ Med 2008 65 482 487 10.1136/oem.2007.032474 18070799

[B53] Relton CL Wilding CS Pearce MS Laffling AJ Jonas PA Lynch SA Tawn EJ Burn J Gene-gene interaction in folate-related genes and risk of neural tube defects in a UK population Journal of medical genetics 2004 41 256 260 10.1136/jmg.2003.010694 15060097 PMC1735724

[B54] Tan CY Chong YS Loganath A Chan YH Ravichandran J Lee CG Chong SS Possible gene-gene interaction of KIR2DL4 with its cognate ligand HLA-G in modulating risk for preeclampsia Reprod Sci 2009 16 1135 1143 10.1177/1933719109342280 19700612

[B55] Jackson HA Carter K Darke C Guttridge MG Ravine D Hutton RD Napier JA Worwood M HFE mutations, iron deficiency and overload in 10,500 blood donors Br J Haematol 2001 114 474 484 10.1046/j.1365-2141.2001.02949.x 11529872

